# Impact of Endodontic and Periodontal Treatments on Oral Health Related Quality of Life and Sleep Quality: A Prospective Study

**DOI:** 10.3390/healthcare14121603

**Published:** 2026-06-06

**Authors:** Oğuz Tavşan, Seden Kara Ongun, Nihan Bulak, Ezgi Can Çekiç, Fatih Karaaslan

**Affiliations:** 1 Department of Endodontics, Faculty of Dentistry, Usak University, Usak 64200, Türkiye; seden.kara@usak.edu.tr (S.K.O.); ezgi.cekic@usak.edu.tr (E.C.Ç.); 2 Department of Periodontology, Faculty of Dentistry, Usak University, Usak 64200, Türkiye; nihan.bulak@usak.edu.tr (N.B.); fatih.karaaslan@usak.edu.tr (F.K.)

**Keywords:** root canal therapy, periodontitis, quality of life, sleep quality, questionnaires

## Abstract

**Background:** Oral and systemic health are strongly associated with individuals’ quality of life and sleep quality, both of which are increasingly recognized as important patient-reported outcomes in dentistry. **Aim:** This prospective longitudinal observational cohort study aimed to compare changes in oral health related quality of life (Oral Health Impact Profile-14; OHIP-14) and sleep quality (Pittsburgh Sleep Quality Index; PSQI) following endodontic and periodontal treatments. **Methods:** A total of 74 patients who received endodontic treatment and 90 who underwent periodontal treatment completed the OHIP-14 and PSQI questionnaires before and three weeks after treatment. Data were analyzed using the Wilcoxon signed-rank test for within-group comparisons and the Mann–Whitney U test on change scores (post−pre) for between-group comparisons, with Holm correction applied for multiple subdomain tests. ANCOVA was additionally performed as an exploratory sensitivity analysis because the homogeneity-of-regression-slopes assumption was violated for both outcomes (*p* < 0.05). **Results:** Both treatments significantly improved OHIP-14 scores and most PSQI components; however, no significant change was observed in sleep medication use in the periodontal group. Functional limitation improved more in the periodontal group, whereas handicap improved more in the endodontic group. Baseline PSQI scores were significantly higher in the endodontic group. PSQI subdomains improved in both groups, with no significant between-group differences after Holm correction. Similarly, no significant differences were found between groups in overall OHIP-14 and PSQI scores. **Conclusions:** Endodontic and periodontal treatments were associated with short-term improvements in individuals’ quality of life and sleep quality. While periodontal treatment may provide greater benefits in certain quality-of-life domains, endodontic treatment may offer advantages in others. Both treatments appear to support improvements in sleep-related outcomes. These findings may contribute to patient-centered clinical decision-making.

## 1. Introduction

Quality of life is defined as “an individual’s perception of their position in life in the context of the culture and value systems in which they live and in relation to their goals, expectations, standards, and concerns” [1]. Rather than being determined solely by health status, it is shaped by a broad range of physical, psychological, social, and environmental influences. Oral health related quality of life (OHRQoL) specifically describes the impact of oral health conditions on daily activities, emotional well-being, and social functioning [2]. Previous research has demonstrated that OHRQoL is associated with various determinants, including demographic characteristics, socioeconomic factors, environmental influences, and oral health status [3].

Studies have demonstrated a positive association between quality of life and various oral health conditions, such as tooth loss [4,5], periodontal diseases [6], endodontic diseases [7,8], and dental anxiety [5]. Oral disorders may adversely affect daily functioning, social interaction, mastication, and aesthetic perception, thereby negatively influencing overall well-being [4]. Consequently, greater emphasis has recently been placed on evaluating how dental treatments influence patient-centered outcomes, particularly oral health related quality of life and sleep quality [7,8].

Endodontic diseases are commonly associated with acute pain, functional impairment, and psychological distress, which may negatively affect oral health related quality of life and sleep quality [9]. Spontaneous and nocturnal pain caused by pulpal and periapical inflammation can interfere with daily functioning and sleep quality. Because endodontic treatment often provides rapid symptom relief, patient-reported outcomes have gained increasing importance in contemporary endodontic research [10]. Periodontal diseases are chronic inflammatory conditions that may adversely affect oral health related quality of life through pain, bleeding, impaired mastication, halitosis, and aesthetic concerns [11]. In addition to their local effects, periodontal inflammation has also been associated with impaired sleep quality and reduced overall well-being [6]. Because periodontal treatment aims to control inflammation and restore oral function, evaluating patient-reported outcomes has become increasingly important in periodontal research [12].

The Oral Health Impact Profile (OHIP) is a widely used instrument developed to evaluate the effects of oral health problems on individuals’ daily lives and well-being. It assesses seven domains related to oral health impacts, including functional limitation, physical pain, psychological discomfort, physical disability, psychological disability, social disability, and handicap. The OHIP-14 represents the abbreviated 14-item version of the original 49-item OHIP questionnaire introduced by Slade and Spencer [13], and subsequently refined and validated by Slade [14]. The Turkish version of the OHIP-14 has previously been translated and validated, with satisfactory reliability and validity demonstrated by Mumcu et al. [15].

Sleep is an essential biological function that plays a major role in maintaining overall physical and mental health. Disturbances in sleep quality or duration have been associated with various adverse effects involving multiple physiological and psychological systems. Epidemiological evidence suggests that approximately 10–30% of adults experience sleep-related problems or poor sleep quality [16].

The Pittsburgh Sleep Quality Index (PSQI) is a commonly used self-reported instrument developed to evaluate sleep quality and sleep-related disturbances in clinical populations. The questionnaire assesses sleep patterns over the previous month using both qualitative and quantitative measures and generates a global score reflecting the severity of sleep-related problems. The PSQI contains 19 items organized into seven component domains: subjective sleep quality, sleep latency, sleep duration, habitual sleep efficiency, sleep disturbances, use of sleeping medication, and daytime dysfunction [17]. The Turkish version of the PSQI has previously been translated and validated, and its reliability and validity have been demonstrated [18].

Previous studies have separately evaluated changes in quality of life and sleep quality following endodontic [8] and periodontal treatments [6]. Recent evidence has also highlighted the increasing importance of patient-reported outcomes in contemporary endodontic and periodontal research [9,10,11,12]. However, comparative evidence regarding the effects of these two treatment modalities on patient-centered outcomes remains limited. Given the distinct clinical characteristics of endodontic and periodontal diseases, including differences in symptom profiles, inflammatory patterns, and treatment responses, these conditions may influence oral health related quality of life and sleep quality in different ways. Therefore, the present study aimed to comparatively evaluate changes in quality of life (OHIP-14) and sleep quality (PSQI) in individuals who underwent endodontic or periodontal treatment. The hypothesis of this study was that endodontic treatment would lead to greater improvements in sleep and quality of life compared to periodontal treatment.

## 2. Methods

This prospective longitudinal observational cohort study was designed to investigate changes in patients’ quality of life and sleep quality following endodontic and periodontal treatments performed by specialist clinicians. All clinical procedures were carried out at the Departments of Endodontics and Periodontology, Faculty of Dentistry, Uşak University. Data collection included a demographic information form (age and sex), the OHIP-14 [15], and the PSQI [17]. The Turkish versions of both questionnaires have previously demonstrated satisfactory validity and reliability [15,18]. All questionnaires were completed through face-to-face interviews.

### 2.1. Ethical Approval

Ethical approval for the study was obtained from the Uşak University Non-Interventional Clinical Research Ethics Committee (decision number: 71-71-01). Written informed consent was obtained from all participants before enrollment in the study. All study procedures were conducted in accordance with the principles of the Declaration of Helsinki and relevant ethical guidelines.

### 2.2. Sample Size

An a priori power analysis was conducted in G*Power 3.1.9.7 for the primary between-group comparison: the change in OHIP-14 total score (post − pre) between the endodontic and periodontal groups, analyzed with an independent two-sample test. Based on previous studies reporting medium between- group effects of non-surgical dental treatment on OHIP-14 total change scores [6,19], a medium effect size (Cohen’s d = 0.50) was assumed, with α = 0.05 (two-tailed) and power = 0.80. The minimum required sample was 128 (64 per group). The final sample exceeded this requirement, yielding an achieved power of 0.86 for the primary comparison.

### 2.3. Inclusion Criteria

To minimize the influence of variable factors such as age, tooth loss, and systemic disease, the following inclusion criteria were established:

- Patients aged between 18 and 65 years.
- Patients without any systemic diseases affecting periodontal tissues.
- Patients who had not used analgesics, anti-inflammatory drugs, or antibiotics recently.
- Non-smokers.
- Patients without any mental disorders.
- Individuals who had not received periodontal treatment within the past six months.

### 2.4. Exclusion Criteria

- Individuals with a diagnosed sleep disorder at baseline

Before participation, all individuals were informed about the aims of the study and the voluntary basis of participation. Participants were also informed that no experimental procedures would be applied during treatment. Confidentiality of the collected data was assured, and all information was used exclusively for scientific purposes. Written informed consent was obtained from all participants before treatment procedures were initiated.

### 2.5. Endodontic and Periodontal Treatment

Participants in both the endodontic and periodontal groups were recruited concurrently between March and June 2023 at the Faculty of Dentistry, Uşak University. A total of 82 patients who agreed to participate underwent dental and medical examinations, and the OHIP-14 and PSQI questionnaires were administered by the researcher during the initial session (T1). The questionnaires at both baseline (T1) and follow-up assessments were administered by the same researcher, whereas all treatment procedures were performed by different clinicians specialized in the relevant fields in order to minimize potential interviewer bias. Participants in the endodontic group were diagnosed with symptomatic irreversible pulpitis based on clinical and radiographic examination findings. Following these assessments, root canal treatments were performed by a different endodontist in a single visit using the RECIPROC Blue (VDW GmbH, Munich, Germany) file system with the VDW Gold endomotor (VDW GmbH, Munich, Germany). During all procedures, 2.5% sodium hypochlorite (NaOCl) was used for irrigation, and the solution was activated using passive ultrasonic irrigation. Following instrumentation and passive ultrasonic irrigation, the final irrigation protocol consisted of sequential application of 2 mL 2.5% NaOCl, 2 mL 17% EDTA, 2 mL 2.5% NaOCl, and distilled water. The root canal system was subsequently obturated using cold lateral compaction with gutta-percha cones (DiaDent, Almere, The Netherlands) in combination with a resin-based sealer (AH Plus; Dentsply DeTrey GmbH, Konstanz, Germany). Permanent restorations were completed using direct composite resin. Three weeks post treatment, patients were recalled for follow-up, at which point endodontic evaluations were conducted, and the OHIP-14 and PSQI questionnaires were re-administered (T2). However, 8 patients did not attend the follow-up session three weeks after treatment was completed; therefore, a total of 74 participants were included in the final analysis.

**Figure 1:**
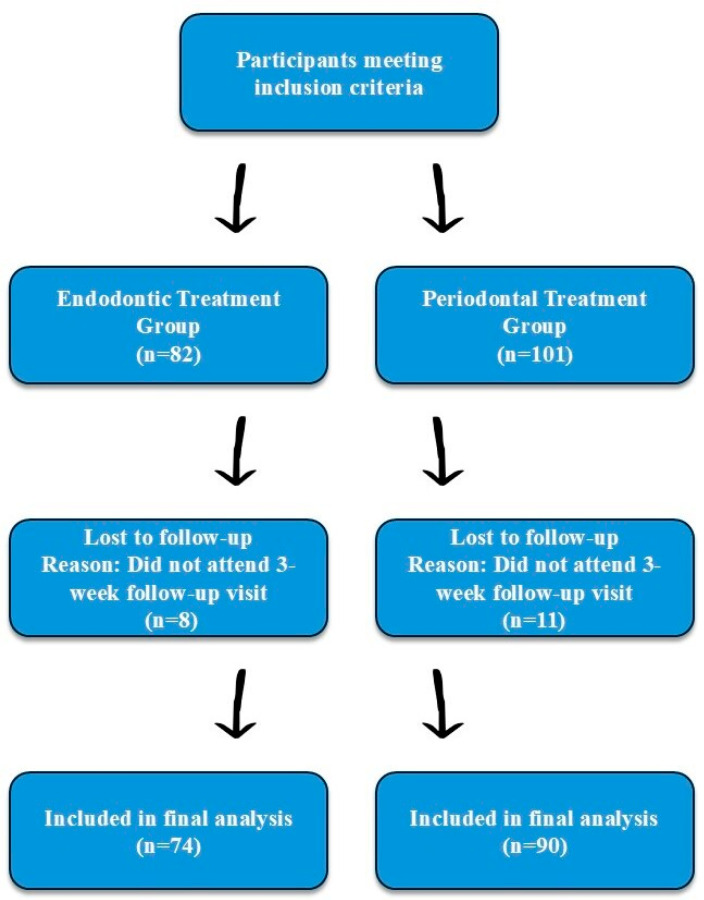
Participant flow diagram for the endodontic and periodontal treatment groups.

For the periodontal group, detailed clinical periodontal examinations were carried out to assess periodontal status. The evaluation included the Plaque Index (PI) described by Silness and Löe [20], Gingival Index (GI) proposed by Löe and Silness [21], probing pocket depth (PPD), and clinical attachment loss (CAL). Periodontitis diagnosis was established on the basis of clinical periodontal findings, particularly PPD and CAL measurements. After completion of the medical and dental history assessments and periodontal examination procedures, the OHIP-14 and PSQI questionnaires were administered by the researcher during the baseline visit (T1). Subsequently, non-surgical periodontal therapy consisting of supragingival scaling and subgingival scaling and root planing was performed by a different clinician. During the first session, patients were provided with oral hygiene education, including demonstrations on brushing techniques, dental flossing, and interdental brush usage using models. Calibration exercises were performed in five periodontitis patients who were not included in the study to assess intra-examiner reliability of the periodontal measurements. The intraclass correlation coefficients (ICC) were 0.88 for probing pocket depth (PPD) and 0.84 for clinical attachment level (CAL), indicating good reliability. After an appropriate healing period of at least three weeks, periodontal parameters were re-evaluated, and the OHIP-14 and PSQI questionnaires were readministered (T2). A three-week follow-up interval was selected to allow sufficient time for the resolution of acute symptoms and initial healing processes following treatment, enabling the early assessment of treatment-related changes in oral health related quality of life and sleep quality. Similar short-term follow-up intervals have been reported in previous studies evaluating patient-reported outcomes following endodontic and periodontal treatments [19,22]. A total of 101 patients agreed to participate; however, 11 patients did not attend the follow-up session three weeks after treatment and were therefore excluded from the study. As a result, 90 participants were included in the final analysis. The flow of participants throughout the study is presented in Figure 1. Only participants who completed both baseline and follow-up assessments were included in the final statistical analyses, and no additional sensitivity analysis was performed for participants lost to follow-up.

### 2.6. Statistical Analysis

Data were analyzed in R (version 4.4.2; R Core Team, 2024) and IBM SPSS Statistics 25. The distribution of continuous variables was assessed with Shapiro–Wilk and Kolmogorov–Smirnov tests; results indicated departure from normality, and non-parametric methods were therefore adopted.

The primary between-group comparison was the Mann–Whitney U test applied to change scores (post−pre) for OHIP-14 and PSQI total and subdomain scores, with the rank-biserial r as the effect size and 95% confidence intervals obtained via Fisher’s z transformation. *p*-values for the seven subdomain tests within each instrument were adjusted for multiple comparisons using the Holm method. Within-group pre–post changes were assessed with the Wilcoxon signed-rank test.

As a sensitivity analysis, ANCOVA was used to model post- treatment total scores with treatment group as a fixed factor and baseline score as a covariate. The homogeneity-of- regression-slopes assumption was tested by including a group × baseline interaction term. Because this assumption was violated for both OHIP-14 and PSQI, ANCOVA estimates are reported as exploratory rather than confirmatory.

Effect sizes were interpreted using conventional thresholds for rank-biserial correlation: r = 0.10 (small), 0.30 (medium), 0.50 (large); partial η^2^ = 0.01 (small), 0.06 (medium), 0.14 (large). All tests were two-tailed; *p* < 0.05 was considered significant.

## 3. Results

The mean age of all participants was 37 years (range: 18–65). Of the participants, 44% were male and 56% were female. Table 1 presents demographic characteristics and baseline scores. No statistically significant differences were observed between the endodontic and periodontal groups in terms of age (U = 3003, *p* = 0.242) or gender distribution (χ^2^ = 0.40, *p* = 0.530). Baseline OHIP-14 total scores did not differ significantly between groups (U = 3044, *p* = 0.303). However, baseline PSQI total scores were significantly higher in the endodontic group (M = 7.64, SD = 3.47) compared with the periodontal group (M = 5.77, SD = 2.81; U = 2316, *p* = 0.001), indicating poorer sleep quality at baseline among patients requiring endodontic treatment.

The OHIP-14 scores before and after treatment for both endodontic and periodontal therapies are presented in Table 2. Significant reductions were observed in all OHIP-14 subdomain scores following both treatments (*p* < 0.05), indicating improvements in quality of life. When comparing changes between treatment types, we found that periodontal treatment resulted in more significant improvements in the domains of functional limitation (*p* = 0.00003), whereas endodontic treatment showed a significantly greater improvement in the handicap domain (*p* = 0.043).

The assumption of homogeneity of regression slopes was tested by including the interaction between baseline OHIP-14 scores and treatment group. The interaction term was statistically significant (F(1,160) = 4.15, *p* = 0.043), indicating a violation of this assumption. For OHIP-14 total change scores, the Mann–Whitney U test (primary between-group comparison) indicated no significant difference between groups (*p* > 0.05), and the magnitude of improvement was comparable between the endodontic and periodontal groups. At the subdomain level, significant between-group differences after Holm correction were observed for Functional Limitation (periodontal > endodontic) and Handicap (endodontic > periodontal). As a sensitivity analysis, ANCOVA using baseline OHIP-14 scores as a covariate is presented only as an exploratory analysis, and the corresponding adjusted estimates are provided in Supplementary Table S1.

The PSQI scores before and after treatment for both groups are shown in Table 3. All PSQI subdomains showed statistically significant improvements in the endodontic group (*p* < 0.05). In the periodontal group, no significant difference was observed in the use of the sleep medication subdomain (*p* > 0.05), whereas all other subdomains demonstrated significant improvement (*p* < 0.05). When comparing PSQI changes between the two treatment groups, no statistically significant differences were observed in any PSQI subdomain after adjustment for multiple comparisons (*p* > 0.05).

For PSQI total scores, the homogeneity of regression slopes assumption was violated (F(1,160) = 8.87, *p* = 0.003). For PSQI total change scores, the Mann–Whitney U test (primary between-group comparison) showed no significant difference between groups (*p* = 0.743), indicating that the magnitude of sleep-quality improvement was comparable between the endodontic and periodontal groups. None of the seven PSQI subdomain comparisons remained significant after Holm correction. As a sensitivity analysis, ANCOVA using baseline PSQI scores as a covariate is presented only as an exploratory analysis, and the corresponding adjusted estimates are provided in Supplementary Table S1.

## 4. Discussion

Endodontic and periodontal treatments not only address localized oral health issues, but they also contribute positively to individuals’ overall quality of life and sleep patterns. Sleep is a complex biological process that plays an important role in maintaining physiological and psychological well-being [23]. It contributes to immune regulation and preservation of inflammatory balance within the body [24]. Sleep disorders have become a major medical and social concern. Due to 24/7 lifestyles and prolonged working hours, reduced sleep quality and duration have become widespread issues as they negatively impact physical health and increase mortality risk [6,25,26]. Although the relationship between endodontic [8] and periodontal diseases [6] and sleep disturbances has been examined separately in the literature, to the best of our knowledge, no previous study has comparatively evaluated changes in sleep and quality of life following these two types of treatment. Despite the growing interest in quality-of-life research, comparative studies evaluating the effects of endodontic and periodontal treatments on patient-centered outcomes remain scarce. Such studies can provide valuable insights into patient expectations and concerns, ultimately contributing to the effectiveness of treatment planning. However, the hypothesis of this study was rejected. No statistically significant between-group differences were observed regarding changes in sleep quality or quality of life.

This study comprehensively evaluated the impact of endodontic treatment on patients’ OHRQoL. The significant reduction observed in post treatment OHIP-14 total scores indicates not only clinical improvement but also an enhancement in psychosocial well-being. Statistically significant improvements across all OHIP-14 subdomains after treatment suggest a general increase in quality of life. Notably, the most significant improvement was observed in the physical pain subdomain, which is consistent with findings in the literature [27]. Studies by Liu et al. [28] and Dugas et al. [7] also reported marked reductions in OHIP-14 scores following endodontic treatment, with the most pronounced improvement in the physical pain domain. Our findings align with those of previous reports.

Consistent with the literature, we found that periodontal treatment had a positive effect on both the OHIP-14 total scores and its subdomains [6,29]. This finding may be related to improvements in gingival health following treatment, which may allow individuals to perform chewing functions more comfortably and perceive improvements in their physical appearance. When comparing endodontic and periodontal treatments, we found that periodontal therapy appeared to be associated with greater improvements on functional limitation. This may be attributed to the nature of periodontal treatment, which targets gingival inflammation and may be associated with improvements in oral function, including chewing and speech [30,31]. Improvements in gingival health may also be associated with improvements in individuals’ self-confidence and social interactions, thereby contributing to overall well-being [32]. Consistent with the existing literature [33], in the endodontic treatment group, the acute nature of preoperative pain and its rapid alleviation following treatment may have contributed to immediate improvements in daily functioning, as suggested by the significant enhancement observed in the Handicap subdomain.

Although clinical research primarily emphasizes statistical significance, evaluating whether treatment-related changes exceed the minimal clinically important difference (MCID) is also important for interpreting patient-reported outcomes [34]. To the best of our knowledge, limited information is available in the literature regarding MCID values for OHIP-14 scores [35]. Previous studies evaluating changes in OHIP-14 scores following dental treatments have suggested an MCID threshold of approximately 0.5 points for the subdomains [34,36]. In the present study, the mean between-group differences observed in the Functional Limitation, Psychological Discomfort, and Handicap subdomains exceeded the MCID values reported in the literature [34,36]. These findings may suggest that some of the observed differences could be clinically perceptible to patients in addition to being statistically significant. However, these findings should still be interpreted cautiously given the observational study design, baseline differences between the groups, and the borderline statistical significance observed in some subdomains.

The PSQI is a widely used and validated questionnaire developed to assess sleep quality [17]. Its inclusion of both qualitative and quantitative components enables a broad evaluation of sleep disturbances and supported a more comprehensive interpretation of the present findings.

High pretreatment PSQI total scores, particularly among patients requiring endodontic treatment, indicated the presence of severe pain and its associated impact on sleep disturbances. Notably, baseline PSQI scores differed significantly between the endodontic and periodontal treatment groups. This discrepancy likely reflects the inherent differences in clinical presentation between the two conditions. Patients undergoing endodontic therapy often present with acute and intense pain [22], which can markedly impair sleep quality, whereas periodontal diseases generally follow a more chronic and frequently asymptomatic course [6]. Therefore, the observed differences in baseline sleep quality may be attributed to the distinct symptom profiles of endodontic and periodontal pathologies. In addition, although no statistically significant age difference was observed between the groups, the relatively broad age range of the study population should be considered when interpreting the comparative findings. Although statistical adjustment was performed to account for baseline PSQI differences, the distinct clinical characteristics of patients requiring endodontic treatment with acute pain conditions and those receiving periodontal therapy for chronic inflammatory disease should be considered when directly comparing treatment effects on sleep outcomes. Differences in baseline disease presentation may also have contributed to the observed variability in patient-reported outcomes between groups. The literature has reported that frequent awakenings due to acute and spontaneous pain negatively impact both sleep latency and sleep duration [37,38]. In our study, root canal treatment was found to effectively eliminate pain and significantly improve PSQI total scores post treatment. Notably, substantial improvements were observed in the PSQI-4 (Habitual Sleep Efficiency) and PSQI-3 (Sleep Duration) subdomains, highlighting the positive influence of endodontic treatment on these key sleep parameters. Evaluations conducted within three weeks after treatment demonstrated improvements in both PSQI subdomains and total scores, suggesting that reductions in spontaneous pain and discomfort following root canal therapy may contribute to improved sleep quality. In patients diagnosed with irreversible pulpitis, achieving a pain-free sleep period post treatment appears to be a significant contributor to this improvement in sleep quality. This improvement may be related to the negative impact of endodontic pathologies on daily quality of life and normal sleep patterns prior to treatment [7]. Relief of spontaneous pain and discomfort following endodontic intervention may facilitate a transition to more regular and higher-quality sleep [8]. In parallel with these findings, a significant reduction in sleep medication use was also observed in the endodontic treatment group. This decrease may be explained by the relief of severe pain and discomfort following successful root canal therapy, which may reduce patients’ reliance on pharmacological support to maintain sleep. In addition to pain relief, the observed improvement in sleep quality following endodontic treatment may also be attributed to biological mechanisms involving systemic inflammation. Endodontic infections are frequently associated with intense pulpal inflammation and may be accompanied by broader inflammatory responses. Therefore, it may be hypothesized that successful endodontic treatment could contribute to improvements in sleep quality not only through pain relief but also through reductions in inflammatory activity. The bidirectional relationship between sleep disturbances and systemic inflammation has been widely discussed in the literature [38]. In this context, endodontic treatment may indirectly improve sleep quality by lowering the systemic inflammatory burden. These mechanisms further support the critical role of endodontic therapy not only in pain management but also in the regulation of sleep-related physiological processes. Our findings are in line with previous studies highlighting the multifactorial pathways through which dental treatment can affect general well-being and sleep regulation [8,10].

Consistent with the literature, this study found that periodontal treatment not only improves gingival health but also enhances individuals’ sleep quality [6,39,40]. Our findings support these previous reports. Specifically, a significant reduction in total PSQI scores was observed following periodontal therapy, indicating a measurable improvement in sleep quality. A previous study reported that individuals with poor sleep quality had a higher risk of developing periodontitis compared with those without sleep disturbances [41]. Periodontal therapy, on the other hand, primarily targets chronic inflammation and facilitates long-term biological recovery, which is consistent with the improvements observed in psychosocial parameters following treatment [6]. These findings emphasize the multifactorial nature of the observed outcomes and highlight how managing chronic oral inflammation may positively influence broader aspects of health, including sleep regulation and emotional well-being. Although differences in sleep duration changes were observed between the groups, these differences did not remain statistically significant after adjustment for multiple comparisons. Nevertheless, both treatment groups demonstrated improvements in sleep-related parameters following treatment, suggesting that reductions in pain and discomfort may contribute to improved sleep quality and daily functioning.

To the best of our knowledge, no study in the field of dentistry has evaluated sleep quality using the PSQI while also determining a MCID. In a study conducted on patients with chronic obstructive pulmonary disease using the PSQI, a reduction of 2 points or more in PSQI scores was considered to represent a clinically meaningful improvement [42]. In the present study, both treatment groups showed reductions greater than 2 points in total PSQI scores, suggesting clinically perceptible short-term improvements within each group. However, the difference in total PSQI change between the endodontic and periodontal groups did not exceed this threshold, suggesting that the magnitude of the between-group difference may be limited from a clinical perspective.

To the best of our knowledge, few studies in the literature have comparatively evaluated the effects of endodontic and periodontal treatments on both quality of life and sleep quality. Considering the close association between oral and general health, understanding how dental treatments influence quality of life and sleep-related outcomes is clinically important. The findings highlight the differences between these two treatment modalities across various parameters, contributing to both clinical practice and patient-centered decision-making processes. Dental treatments not only influence local oral health but also impact individuals’ overall quality of life and psychophysiological balance. In this regard, future studies should consider conducting long-term evaluations with larger and more diverse samples, considering factors such as age groups, history of systemic diseases, and sociodemographic variables. Such efforts may further support the development of patient-centered treatment planning. Nonetheless, this study has certain limitations. Due to the open-label nature of the study design, blinding of participants and outcome assessors was not feasible, and this should be considered an inherent methodological limitation. The compared patient groups consisted of individuals who received either endodontic or periodontal treatment exclusively. Because this was not a randomized or matched comparative study, the findings should not be interpreted as evidence of direct superiority between treatment modalities. The distinct biological and clinical characteristics of endodontic and periodontal diseases, including differences in disease severity, clinical extent, and pain status, should be considered when directly comparing treatment-related patient-reported outcomes. Although standardized treatment protocols and strict inclusion/exclusion criteria were applied to reduce variability, these factors may still have influenced the observed findings. Future research should investigate changes in sleep and quality of life in patients receiving both treatments concurrently. One of the main limitations of this study is the short follow-up period, which was limited to three weeks. Although this duration is consistent with several previously published short-term assessments, it may be insufficient to evaluate sustained effects, long-term outcomes, delayed improvements, or potential relapses. Periodontal tissue healing and clinical remodeling generally continue for several weeks following treatment [43]. Therefore, the three-week follow-up period used in the present study may not have been sufficient to fully reflect the effects of periodontal therapy on oral health related quality of life and sleep quality. Because sleep and quality-of-life outcomes were evaluated only in the short term, the long-term effects of the treatments cannot be clearly determined. Future studies with extended follow-up periods are needed to provide a more comprehensive evaluation of the long-term outcomes of dental treatments.

One of the limitations of this study is sleep and quality of life are multifaceted constructs influenced by various external factors, such as lifestyle, occupational and personal stress levels, and psychological status. In addition, baseline dental anxiety or pain catastrophizing may also influence sleep quality; however, these psychological variables were not evaluated in the present study and should be considered potential unmeasured confounders. These factors were not controlled for in the present study, and it should be acknowledged that some of the observed changes may have resulted from nontreatment-related variables. Furthermore, because the homogeneity-of-regression-slopes assumption was violated for both OHIP-14 and PSQI total scores, ANCOVA findings were treated as exploratory, and primary between-group inferences were based on non-parametric analyses of change scores. Accordingly, residual confounding related to baseline differences in disease presentation, particularly between acute endodontic and chronic periodontal conditions, cannot be fully excluded.

## 5. Conclusions

This study provides a unique contribution by comparatively evaluating short-term changes in quality of life and sleep quality following endodontic and periodontal treatments. Both treatment approaches were associated with improvements in patient-reported outcomes, including oral health related quality of life and sleep quality. Improvements in sleep-related parameters following endodontic treatment may be linked to the rapid relief of spontaneous pain commonly associated with endodontic conditions. In contrast, periodontal treatment was associated with greater improvement in functional limitation, possibly reflecting the beneficial effects of periodontal therapy on oral function and daily activities. However, these comparative findings should be interpreted cautiously given the distinct clinical characteristics and baseline differences between the study populations.

## Figures and Tables

**Table 1:** Demographic and baseline characteristics of the study groups.

Variable	Endodontic Treatment (*n* = 74)	Periodontal Treatment (*n* = 90)	Test Statistic	*p*
Age (years), M ± SD	36.3 ± 15.2	37.5 ± 7.1	*U* = 3003	0.242
Gender (Male/Female)	30/44	42/48	χ^2^ = 0.40	0.530
Baseline OHIP-14, M ± SD	18.49 ± 10.93	19.87 ± 8.20	*U* = 3044	0.303
Baseline PSQI, M ± SD	7.64 ± 3.47	5.77 ± 2.81	*U* = 2316	0.001 **

Note. M = mean; SD = standard deviation. Mann–Whitney U test for continuous variables; chi-square for gender. ** *p* < 0.01. Values in bold indicate statistical significance.

**Table 2:** The Oral Health Impact Profile-14 (OHIP-14) Scores.

OHIP-14	Endodontic Treatment						Periodontal Treatment					
	Mean (Pre)	Mean (Post)	Difference	*p* (Within)	r	Mean (Pre)	Mean (Post)	Difference	*p* (Within)	r	*p* (Between)	*p*.adj (Holm)
**Functional** **Limitation**	1.41	1.00	−0.405	0.011	0.30	3.244	1.933	−1.311	<0.001	0.81	<0.001	**0** .00003 *
**Physical Pain**	4.54	2.43	−2.108	<0.001	0.68	3.178	1.844	−1.333	<0.001	0.81	0.052	0.839
**Psychological Discomfort**	3.35	2.68	−0.676	0.038	0.24	3.244	1.867	−1.378	<0.001	0.74	0.003	0.054
**Physical** **Disability**	2.22	1.22	−1.000	<0.001	0.52	3.111	1.978	−1.133	<0.001	0.71	0.791	1.000
**Psychological Disability**	2.19	1.24	−0.946	<0.001	0.49	3.044	1.822	−1.222	<0.001	0.71	0.075	1.000
**Social** **Disability**	2.38	1.54	−0.838	<0.001	0.53	2.578	1.489	−1.089	<0.001	0.73	0.380	1.000
**Handicap**	2.41	1.11	−1.297	<0.001	0.55	1.467	0.933	−0.533	<0.001	0.58	0.003	**0** .043 *
**Total OHIP**	18.49	11.22	−7.270	<0.001	0.69	19.867	11.867	−8.000	<0.001	0.82	0.707	1.000

* *p* (between) = Indicates comparison of endodontic and periodontal treatment by Mann–Whitney U Test; *p* (within) = Wilcoxon signed-rank (within-group); r = effect size; Holm-adjusted *p*-values were applied to control for the increased risk of Type I error. Values in bold indicate statistical significance.

**Table 3:** Pittsburgh Sleep Quality Index (PSQI) Scores.

PSQI	Endodontic Treatment						Periodontal Treatment					
	Mean (Pre)	Mean (Post)	Difference	*p* (Within)	r	Mean (Pre)	Mean (Post)	Difference	*p* (Within)	r	*p* (Between)	*p*.adj (Holm)
**Subjective Sleep Quality**	1.324	0.973	−0.351	0.002	0.37	1.022	0.600	−0.422	<0.001	0.62	0.636	1.000
**Sleep** **Latency**	1.649	1.162	−0.486	<0.001	0.43	0.956	0.444	−0.511	<0.001	0.66	0.893	1.000
**Sleep** **Duration**	0.959	0.446	−0.513	<0.001	0.61	0.919	0.541	−0.378	0.003	0.36	0.048	0.073
**Habitual Sleep** **Efficiency**	0.432	0.081	−0.351	<0.001	0.48	0.800	0.400	−0.400	<0.001	0.54	0.462	1.000
**Sleep** **Disturbances**	1.784	1.243	−0.541	<0.001	0.51	0.800	0.422	−0.378	<0.001	0.58	0.086	1.000
**Use of** **Sleeping** **Medication**	0.297	0.027	−0.270	0.008	0.31	0.267	0.244	−0.022	0.717	0.04	0.106	1.000
**Daytime Dysfunction**	1.189	0.703	−0.486	<0.001	0.47	0.867	0.311	−0.556	<0.001	0.66	0.379	1.000
**Total PSQI**	7.634	4.635	−2.998	<0.001	0.68	5.631	2.962	−2.667	<0.001	0.83	0.832	1.000

*p* (between) = Indicates comparison of endodontic and periodontal treatment by Mann-Whitney U Test; *p* (within) = Wilcoxon signed-rank (within-group); r = effect size; Holm-adjusted *p*-values were applied to control for the increased risk of Type I error.

## Data Availability

The datasets generated and/or analyzed during the current study are not publicly available but are available from the corresponding author upon reasonable request. The data underlying this study are available from the corresponding author on reasonable request. However, the datasets are not publicly available due to privacy and confidentiality considerations, as they contain participant-related information (e.g., questionnaire responses and demographic data).

## Supplementary Materials

The following supporting information can be downloaded at: https://www.mdpi.com/article/10.3390/healthcare14121603/s1, Table S1: ANCOVA results for post-treatment OHIP-14 and PSQI total scores adjusted for baseline values.
