# Supplementary material for: Impact of Endodontic and Periodontal Treatments on Oral Health Related Quality of Life and Sleep Quality: A Prospective Study
Source: Healthcare (Basel). 2026 Jun 6;14(12):1603. doi: 10.3390/healthcare14121603 (PMC13299726; doi:10.3390/healthcare14121603)
Supplement: Supplementary file 1 [file healthcare-14-01603-s001.zip › healthcare-4288327-supplementary.pdf]

Supplementary Table S1. ANCOVA results for post-treatment OHIP-14 and PSQI total scores adjusted for baseline values

**Outcome**	**Slopes Test**	**F(1, 161)**	**p**	**η^2_p**	**Adj. M Endo**	**Adj. M Perio**	**Interpret.**
OHIP-14 Total	$F = 4.15$, $p = .043$†	< 0.01	.982	< .001	11.58	11.56	Negligible
PSQI Total	$F = 8.87$, $p = .003$†	13.40	**< .001*** **	.077	4.27	3.26	Medium

Note. **Adj. M = adjusted (estimated marginal) mean controlling for baseline scores. † Homogeneity of regression slopes assumption violated; interpret with caution. *** $p < .001$.**
